# Systematic review of the impact of heatwaves on health service demand in Australia

**DOI:** 10.1186/s12913-022-08341-3

**Published:** 2022-07-28

**Authors:** Hannah Mason, Jemma C King, Amy E Peden, Richard C Franklin

**Affiliations:** 1 Discipline of Public Health and Tropical Medicine, James Cook University, Townsville, Queensland 4811 Australia; 2 School of Population Health, Faculty of Medicine, University of New South Wales, Sydney, New South Wales Australia

**Keywords:** Extreme heat, Climate change, Health system, Australia, Disaster health

## Abstract

**Objectives:**

Heatwaves have been linked to increased levels of health service demand in Australia. This systematic literature review aimed to explore health service demand during Australian heatwaves for hospital admissions, emergency department presentations, ambulance call-outs, and risk of mortality.

**Study design:**

A systematic review to explore peer-reviewed heatwave literature published from 2000 to 2020.

**Data sources:**

Articles were reviewed from six databases (MEDLINE, Scopus, Web of Science, PsychINFO, ProQuest, Science Direct). Search terms included: heatwave, extreme heat, ambulance, emergency department, and hospital. Studies were included if they explored heat for a period of two or more consecutive days. Studies were excluded if they did not define a threshold for extreme heat or if they explored data only from workers compensation claims and major events.

**Data synthesis:**

This review was prospectively registered with PROSPERO (#CRD42021227395). Forty-five papers were included in the final review following full-text screening. Following a quality assessment using the GRADE approach, data were extracted to a spreadsheet and compared. Significant increases in mortality, as well as hospital, emergency, and ambulance demand, were found across Australia during heatwave periods. Admissions for cardiovascular, renal, respiratory, mental and behavioural conditions exhibited increases during heatwaves. The most vulnerable groups during heatwaves were children (< 18 years) and the elderly (60+).

**Conclusions:**

Heatwaves in Australia will continue to increase in duration and frequency due to the effects of climate change. Health planning is essential at the community, state, and federal levels to mitigate the impacts of heatwaves on health and health service delivery especially for vulnerable populations. However, understanding the true impact of heatwaves on health service demand is complicated by differing definitions and methodology in the literature. The Excess Heat Factor (EHF) is the preferred approach to defining heatwaves given its consideration of local climate variability and acclimatisation. Future research should explore evidence-based and spatially relevant heatwave prevention programs. An enhanced understanding of heatwave health impacts including service demand will inform the development of such programs which are necessary to promote population and health system resilience.

**Supplementary Information:**

The online version contains supplementary material available at 10.1186/s12913-022-08341-3.

## Introduction

The number of climate disasters per annum are increasing globally, as is the length and severity of these events [1, 2]. The World Health Organization (WHO) recognises that the number of climate-related disasters has tripled in the last 60 years, resulting in over 60,000 deaths per annum [1]. Of particular concern are heatwaves, as global temperatures continue to rise at record breaking rates [3]. Major heatwave events across the globe, as seen in Europe in 2003, Russia in 2010, and Asia in 2015, and more recently Australia’s Black Summer in 2019–20, have resulted in mass casualties and strain on health systems [4–7]. Climate change is exacerbating the intensity and length of heatwaves, and mortality will continue to increase in the future in the absence of adequate leadership, preparedness, and prevention [8, 9]. The occurrence of record-breaking heat periods is five times higher than what would be expected without the influence of climate change [9]. Approximately 30% of the world’s population experience at least 20 days per year in which the deadly threshold of extreme heat is reached (i.e. the threshold of temperature and humidity projected to cause death) [2, 10]. If current carbon dioxide (CO_2_) emissions continue without intervention, it is expected that this will increase to 74% [2, 10]. According to the WHO, there were more than 166,000 deaths due to heatwaves between 1998 and 2017 [11].

Internationally there is limited agreement on the specific parameters that must be in play for a significant heat event to be considered a heatwave [12]. In comparison with other disasters such as earthquakes and cyclones, heat events occur gradually rather than causing sudden and violent death, and therefore it is difficult to determine a beginning and end point [12]. Heatwaves are classified as a meteorological disaster under the category of extreme temperature [12, 13]. Put simply, the key components are that the temperature increases rapidly, remains high for an extended period of time and then drops off again. The use of a proportional increase in temperature, ambient temperature measurements, the duration that the temperature must be sustained and the ‘extremeness’ of the temperature are key contention points for the various definitions [14]. The central challenge with not having a standard definition for heatwaves is that it becomes difficult to ascertain whether a health presentation or death has occurred within, or due to a period of extreme heat [15]. This lack of standardisation results in further implications for documentation of cases and public health planning [15].

In Australia, the growing number of recorded heatwaves and extreme heat events each year is a health concern, as heatwaves kill more people than any other natural hazard [16, 17]. Projections for future hot weather in Australia echo global trends, as increasing extreme heat has been observed across Australia [6, 18]. Australia has experienced a consistent increase in amplitude, magnitude, frequency, and duration of heatwaves which have been attributed to the effects of climate change [9]. Studying heatwaves in Australia is complex and challenging due to climate variation between and within states [19]. Given this variability and subsequent implications for resident acclimatisation, the use of a heatwave definition that acknowledges temperature differences and spatial factors between locations is appropriate [20]. Most Australian jurisdictions have adopted emergency heat management plans which include various formulas to predict when heat events will occur [7, 21]. The formulas utilised in these plans, however, vary between states, leading to substantial differences in resultant health service activation to cope with demand during these periods [21].

To develop effective planning and prevention strategies, it is necessary to explore the full spectrum of heatwave-vulnerability drivers [22]. Determining the pathology of heatwave morbidity and mortality is a proxy of many factors not just temperature but includes medications, co-morbidities, physiology and age [23, 24]. Heat-health relationships vary across communities in Australia, with differing magnitudes of heatwave morbidity and mortality both amongst and within regions [18, 24]. Increases in health service demand are expected particularly from vulnerable groups [9, 18, 25]. Poor health, income, social isolation, and the built-environment have been identified as key intersecting factors which influence heat-health risk [9, 24, 26].

As temperatures continue to increase in Australia, adverse health events during periods of extreme heat will increase demand on the health system including increases for ambulance services, emergency departments, and hospital admissions [6, 17]. Estimations of future costs associated with extreme heat across Australia are not well explored, however Tong et al. [27] projected that heat attributable hospital costs in Perth, Western Australia alone will increase to 125.8–129.1 million AUD within the next decade.

The purpose of this review is to provide information to prepare health services for heat events, which is a necessary aspect of public health planning [9, 17]. Thus, this systematic review aims to:

- Describe what is known about the burden of heat on health service demand in Australia from the literature,
- Describe what current indicators are used for measuring the impact of heat on health services and specifically what health conditions, ages, and other factors increase risk,
- Describe the current peer-reviewed evidence for the preparation of health services for heat, and
- Identify gaps in the literature for future research.

## Materials and methods

A systematic review of peer-reviewed literature published between 1 January 2000 and 31 December 2020 was conducted according to the preferred reporting items for systematic review and meta-analysis protocol (PRISMA) [28] and prospectively registered with PROSPERO (#CRD42021227395) [29]. The aims of this study were modified from the time of PROSPERO registration to explore only Australian data. The PICO (Population, Interest Area, Comparator, Outcome(s)) framework was used to structure this systematic review [30]. Our review questions were:

- How do heatwaves impact demand on health systems in Australia?
- What indicator(s) are used for measuring the impact of heat on demand for health services?
- What medical condition(s), age(s), and other factor(s) increase risk of heat-related presentations to medical care?

### Systematic review framework

In order to answer these questions, the researchers explored the impact of heatwave or a prolonged heat period (henceforth referred to as a heatwave) on health service demand in Australia (Population and Interest Area). The primary outcomes were the relative risk, odds ratio or risk difference of ambulance call outs, presentation to emergency department (ED), hospital separations or mortality (Outcome) during a heatwave compared to non-heatwave periods (Comparator). A secondary outcome of interest was specific medical conditions implicated in heatwave-related health service impact (Outcome). Also of interest was variance in definitions used for heatwave.

### Definitions

#### Heatwave

A period of two or more consecutive days of extreme heat above a specified temperature threshold or percentile threshold.

#### Excess heat factor (EHF)

A measure to indicate the severity of heatwave events which incorporates historical and acclimatization factors using a three day average temperature for a given location. See Nairn & Fawcett [20] for full methodology.

### Search strategy

Peer-reviewed primary literature published between 1 January 2000 and 31 December 2020 was identified by searching MEDLINE, Scopus, Web of Science, PsychINFO, ProQuest and ScienceDirect databases. Searches were run from 11th–15th January 2021 with limits applied to English language and date range (published date 2000–2020). The date range for publications was limited from 2000 onwards to capture a 20-year period where heatwaves have increased, have been recognized by governments and health organisations as an issue, and mitigation strategies have been implemented. Key search terms included heatwave, extreme heat, ambulance, hospital, and emergency department. Studies were included if they explored health outcomes for two or more consecutive days of extreme heat in Australia. There were no restrictions set for study period. Studies which did not define a threshold for “extreme heat” or did not define “heatwaves” were excluded. Studies that explored data from workers compensation claims or from major events (marathons, music festivals, religious festivals, etc.) were excluded. The full search strategy can be found in Supplementary File 1.

### Study selection

Dual screening of title and abstract was undertaken independently by authors (HM, JK and AP). Author RF resolved any conflicts. Single screening of full text studies was conducted independently by authors, with a random double check of 20% (HM, JK, AP and RF).

### Data extraction and analysis

Data from each of the included studies were extracted by one of four authors (HM, JK, AP, RF). A data extraction form in Microsoft Excel was used (see Supplementary File 2- Excel file). Data extracted were: author, published year, years study covers, months covered, study location, heatwave definition(s), reference in paper for heat definition, age groups covered by paper, age groups by which data could be disaggregated, health service explored (i.e., ambulance attendance, ED presentation, hospital separation or mortality), medical conditions (coded as overall, cardiovascular, renal, respiratory, diabetes, asthma, chronic obstructive pulmonary disease [COPD], diarrhoea, mental health, Alzheimer’s Disease, heat-related injury, electrolyte imbalance, dehydration, nervous system, and neoplasm), and risk factors (classified as age, socio-economic status [SES], sex, Aboriginal and Torres Strait Islander status, rurality, coastal dwelling, holiday, living alone, air-conditioning in bedroom, social factors, pollutant exposure, living in aged care, prior fall, number of comorbidities, no private health insurance, having an emergency button, using refreshments, education, receiving community supports, and urban greenspace). Medical conditions were later grouped into broader disease categories (Table 2). For data extraction, in studies where multiple definitions of heatwave were explored, data were extracted from the studies in which the Excess Heat Factor (EHF) was used, or if EHF was not used from the definition which used the parameters of: 95th percentile of temperature for two or more consecutive days. Where the EHF was used, and data were stratified into heatwave intensity (low intensity, severe, and extreme), data from severe heatwaves were used. Severe heatwaves were selected as the comparator with the assumption that they would have a greater impact than low intensity heatwaves and occur more frequently than extreme heatwaves.

The level of significance was set at *p* < .05. Data were organised from the extraction file to construct tables for the impact of heatwaves on presentations to health services, the impact of heatwaves on presentations of medical conditions, and risk factors for heatwave morbidity and mortality. Risk factors were further categorized into the climate vulnerability categories of exposure (i.e. degree of exposure to heatwaves due to location), sensitivity (i.e. degree of impact of heatwave on vulnerable people, for example those with cardiac conditions more likely to be impacted), and adaptive capacity (i.e. ability to mitigate harm from heatwaves) [46].

Risk of bias and quality assessment was conducted using the Strengthening the Reporting of Observational Studies in Epidemiology (STROBE) statement [47]. As the studies in this review were of an observational nature, STROBE was determined to be the most appropriate quality assessment tool [47]. The risk of bias assessment was conducted by two authors independently (HM, RF) and then double checked by a third author (AP or JK). The assessment was modified to the items that were appropriate and applicable for each study, and raw scores were converted into a percentage out of 100 (Supplementary File 3). The quality of evidence across all studies was assessed using the Grading of Recommendations Assessment, Development, and Evaluation (GRADE) approach [48]. The body of evidence was initially rated as moderate and then downgraded based on five possible factors (risk of bias, indirectness, inconsistency, imprecision and publication bias) and upgraded based on three possible factors (size of effect, dose response pattern, possibility of confounding) [48, 49].

## Results

**Fig. 1:**
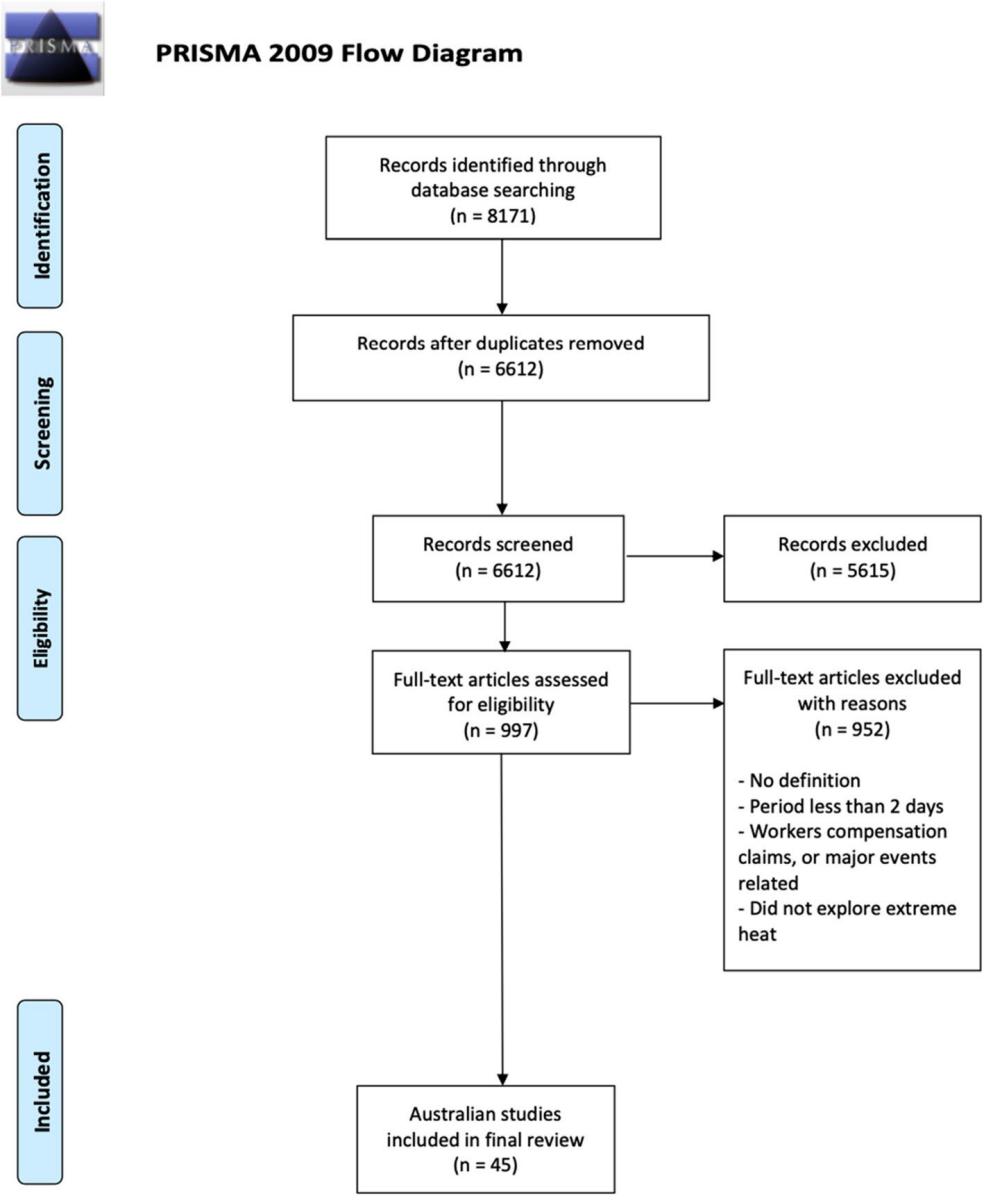
PRISMA flow chart

A total of 6612 articles were identified after removing duplicates. Initial title and abstract screening yielded 997 articles, which were reduced to a final inclusion of 50 studies after full text review. Figure 1 summarises the results of the article search and selection process in a modified PRISMA [28]. Forty-five studies provided numerical data for data extraction, the remaining five studies are summarized in Supplementary File 4. Results of the STROBE analysis indicated that the quality of the papers was good, with two papers scoring from 60 to 69%, 23 papers scoring from 70 to 79%, 16 papers scoring from 80 to 89%, and four papers scoring from 90 to 100% (Supplementary File 3).

The overall rating of the quality of evidence was of moderate quality. It was concluded by both reviewers that there was no substantial risk of bias across the body of evidence included in the review (0). The evidence was directly comparable to the question of interest (PICOT) (0). There were differences in the magnitude effect across the studies. The reviewers agreed that these differences were likely due to variation in methodology (heatwave definition, measures of effect, conditions explored), and as such was downgraded (− 1). The majority of the studies included in the review utilized population-based data and no studies were included with a small number of participants, but there were some wide confidence intervals. The quality of evidence was downgraded for imprecision (− 1). It was determined that there was no substantial risk of publication bias (0). Though unpublished studies and grey literature were not included, the search was comprehensive and studies were not uniformly small or sponsored by industry. Effect sizes were considered small to medium for overall heatwave morbidity and mortality, with larger effect sizes seen for condition-specific morbidity and mortality. Confounding alone was unlikely to explain associations of large magnitudes, and quality of evidence was upgraded (1). Dose response was determined for studies which used multiple definitions of heatwaves, with higher heatwave thresholds exhibiting larger effect sizes (1). There was no evidence that results were influenced by residual confounding variables, and thus quality of evidence was not upgraded (0). (Table 1).

**Table 1:** Summary of findings for literature quality of evidence

Quality factor	Rating	Rationale
Downgrade		
Risk of bias across studies	0	There is no substantial risk of bias across the body of evidence included in the review
Indirectness	0	The studies were directly related to the question of interest (PICOT)
Inconsistency	−1	Studies were not always consistent regarding the magnitude and direction of effect of heatwaves.
Imprecision	−1	Studies included sufficient participants. Some wide confidence intervals occurred specifically in the condition-specific data.
Publication bias	0	There was no substantial risk of publication bias across the body of evidence included in the review.
Upgrade		
Large magnitude of effect	1	Large magnitude of effect for condition specific studies. Results were unlikely to be explained by confounding alone.
Dose response	1	Dose-response relationship evident when multiple definitions of heatwaves were used.
Confounding minimizes effect	0	No evidence that residual confounding underestimated the effect.

**Fig. 2:**
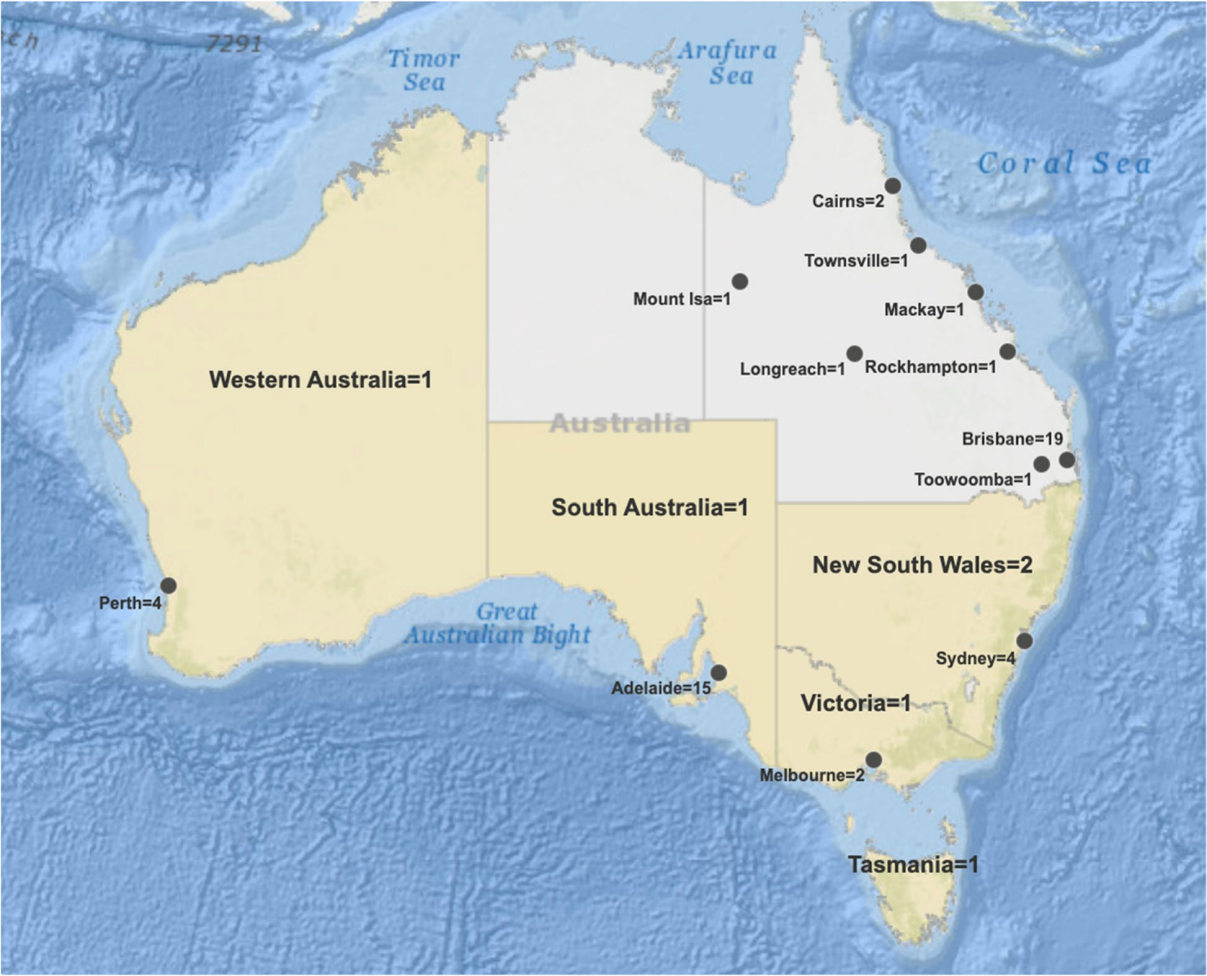
Map of heatwave study locations across Australia

Of the 45 papers selected for extraction, 17 were from Queensland [38–41, 50–62], 15 were from South Australia [32, 43, 44, 63–74], four were from Western Australia [31, 34, 35, 42], four were from New South Wales [36, 37, 75, 76], two were from Victoria [77, 78], one was from Tasmania [33], and two used multi-location data [16, 45]. Thirty-nine studies analysed independent or multi-city data, [16, 31, 32, 34, 37–45, 50–70, 72–74, 76, 77] and six studies conducted a state-wide analysis, as indicated after the state name (Fig. 2) [33, 35, 36, 71, 75, 78]. There were no articles which utilized Australia-wide data. The years 1998 to 2016 were covered in the reported data.

Heatwave health outcomes were reported by using seven different epidemiological measures: 15 reported relative risk (RR) [31, 34, 35, 37, 39, 40, 42, 43, 45, 51, 52, 59–61], 12 reported odds ratios (OR) [33, 38, 41, 54, 66, 67, 72–76, 78], nine reported incidence rate ratios (IRR), [32, 36, 44, 63–65, 69–71] six reported crude increases in admissions [55–58, 68, 77], two reported change in admissions as a percent [16, 53], and one reported years of life lost (YLL) [50].

As expected, heatwave definitions varied in each study, with 16 studies using a combination of temperature limit (e.g. 90th, 95th, 99th percentile) and duration (e.g. 2+, 3+, 4+ days), [32, 38, 39, 51, 53, 54, 61, 64, 65, 68–70, 72–74, 78] eight studies using the EHF [33–36, 42, 43, 63, 71], two studies using the three day average above a temperature threshold [75, 77], one using a Bureau of Meteorology (BOM) identified heatwave period [37], and 18 reporting multiple definitions [16, 31, 40, 41, 44, 45, 50, 52, 55–60, 62, 66, 67, 76].

Overall, increases in hospital admissions, ED presentations, ambulance call outs, and mortality were present during heatwave periods in comparison to non-heatwave periods (Table 2). Individuals who had a significantly higher risk of presenting to a health service during heatwave periods were those with cardiovascular, renal, respiratory, and neurological, mental, and behavioural medical conditions (Table 3).

**Table 2:** Heatwave impacts on health service demand (*n* = 16)

Location/Data Source	Heatwave Definition	Study type	Effect size (95% CI)	Reference
Hospital Admissions				
Perth, Western Australia	EHF	Retrospective population-based	RR = 1.58 (1.18, 2.11) *	Scalley et al. (2015) [31]
Adelaide, South Australia	≥35 °C, 3+ days	Case-series analysis	IRR = 1.07 (0.99, 1.16)	Nitschke et al. (2007) [32]
Emergency Department Presentations				
Tasmania	EHF	Case-crossover analysis	OR = 1.05 (1.01, 1.09) *	Campbell et al. (2019) [33]
Perth. Western Australia	EHF	Population-based time series	OR = 1.05 (1.05, 1.06) *	Patel et al. (2019b) [34]
Perth, Western Australia	EHF	Retrospective population-based	RR = 1.04 (1.04, 1.05) *	Scalley et al. (2015) [31]
Western Australia	EHF_Severe/extreme_	Time series analysis	RR = 1.05 (1.04, 1.06) *	Xiao et al. (2017) [35]
New South Wales	EHF_Intense_	Time series analysis	IRR = 1.04 (1.02, 1.05) *	Jegasothy et al. (2017) [36]
Sydney, New South Wales	BOM identified	Time series analysis	RR = 1.02 (1.01, 1.03) *	Schaffer et al. (2012) [37]
Brisbane, Queensland	> 37 °C, 2+ days	Case-crossover analysis	OR = 1.15 (1.08, 1.24) *	Wang et al. (2012) [38]
Brisbane, Queensland	> 37 °C, 2+ days	Time-stratified case-crossover analysis	OR = 1.14 (1.06, 1.23) *	Tong et al. (2012) [39]
Brisbane, Queensland	≥95th percentile, 2+ days	Time series analysis	RR = 1.10 (1.08, 1.13) *	Tong et al. (2014) [40]
Brisbane, Queensland	≥95th percentile, 3+ days	Case-crossover analysis	OR = 1.04 (1.02, 1.06) *	Tong et al. (2010) [41]
Ambulance call outs				
Perth, Western Australia	EHF	Population-based time series	RR = 1.02 (1.01, 1.02) *	Patel et al. (2019a) [42]
Sydney, New South Wales	EHF	Time-series analysis	RR = 1.14 (1.11, 1.16) *	Schaffer et al. (2012) [37]
New South Wales	EHF_Intense_	Time series analysis	IRR = 1.05 (1.04, 1.06) *	Jegasothy et al. (2017) [36]
Adelaide, South Australia	EHF_Intense_	Case-crossover analysis	RR = 1.21 (0.81, 1.81)	Varghese et al. (2019) [43]
Adelaide, South Australia	BOM identified	Retrospective population-based	RR = 1.11 (1.08, 1.13) *	Williams et al. (2011) [44]
Adelaide, South Australia	≥35 °C, 3+ days	Case-series analysis	IRR = 1.04 (1.01, 1.07) *	Nitschke et al. (2007) [32]
Mortality				
Sydney, New South Wales	EHF	Time-series analysis	RR = 1.13 (1.06, 1.22) *	Schaffer et al. (2012) [37]
New South Wales	EHF_Intense_	Time series analysis	IRR = 1.02 (1.01, 1.04) *	Jegasothy et al. (2017) [36]
Adelaide, South Australia	BOM identified	Retrospective population-based	IRR = 1.06 (1.00, 1.11)*	Williams et al. (2011) [44]
Mortality Con’t				
Adelaide, South Australia	≥35 °C, 3+ days	Case-series analysis	IRR = 0.95 (0.90, 1.01)	Nitschke et al. (2007) [32]
Brisbane, Queensland	> 37 °C, 2+ days	Case-crossover analysis	OR = 1.46 (1.21, 1.77) *	Wang et al. (2012) [38]
Brisbane, Queensland	> 37 °C, 2+ days	Time-stratified case-crossover analysis	RR = 1.92 (1.40, 2.11) *	Tong et al. (2012) [39]
Brisbane, Queensland	>95th percentile, 2+ days	Time series analysis	RR = 1.05 (1.03, 1.08) *	Wang et al. (2015) [45]
Melbourne, Victoria	>95th percentile, 2+ days	Time series analysis	RR = 1.03 (1.01, 1.05) *	Wang et al. (2015) [45]
Sydney, New South Wales	>95th percentile, 2+ days	Time series analysis	RR = 1.04 (1.02, 1.06) *	Wang et al. (2015) [45]
Brisbane, Queensland	≥95th percentile, 2+ days	Time series analysis	RR = 1.17 (1.10, 1.25) *	Tong et al. (2014) [40]
Brisbane, Queensland	≥95th percentile, 3+ days	Case-crossover analysis	OR = 1.10 (1.03, 1.18) *	Tong et al. (2010) [41]

Abbreviations: *OR* Odds Ratio, *RR* Relative Risk, *IRR* Incident Rate Ratio, *EHF *Excess Heat Factor, *BOM *Bureau of Meteorology

* denotes statistically significant values at *p* < 0.05

**Table 3:** Significant effects of heatwaves on presentations in Australia by condition (*n* = 21)

	Medical Condition	Ages
**Hospital Admissions**	Cardiovascular related	All ages [50, 74] 35+ years [77], 65–74 years [32]
	Renal related	All ages [32, 35, 63, 65, 69, 73]
	Nervous system, mental and behavioral	All ages [32, 64, 69, 74], 35+ years [62],
	Heat related	All ages [69]
**Emergency Department**	Cardiovascular related	All ages [51, 75]
	Renal related	All ages [38, 63, 69, 70, 75], 0–14 years [54]
	Respiratory related	All ages [51, 75]
	Nervous system, mental and behavioral	All ages [61, 69, 75]
	Endocrine, nutritional, and metabolic	All ages [61]
	Diseases of the genitourinary system	All ages [61]
	Neoplasm	All ages [75]
	Heat related	All ages [52, 69, 70], 0–14 years [52], 15–64 [52], 65–74 [52], 75 + [52]
**Ambulance Call Outs**	Cardiovascular	All ages [53, 69]
	Respiratory	All ages [53, 69], 5–14 years [69], 15–64 years [69]
	Nervous system, mental and behavioral	65–74 [69]
**Mortality**	Cardiovascular	All ages [38, 45, 72]
	Nervous system, mental and behavioral	All ages [64], 35+ years [62]
	Diabetes	75+ years [38]

See Supplementary File 5 for detailed report

The most common risk factor for heatwave presentations and mortality was advanced age (60+ years) (*n* = 16), followed by low socioeconomic status (*n* = 4), under 18 years of age (*n* = 3), living alone (*n* = 2), pollutant exposure (*n* = 2), receiving community support (*n* = 1), not having private health insurance (*n* = 1), experiencing a prior fall (*n* = 1), Aboriginal and Torres Strait Islander status (*n* = 1), holiday periods (*n* = 1), coastal dwelling (*n* = 1), and urban dwelling (*n* = 1). Protective factors were having air conditioning in the bedroom (*n* = 2), having more social activities (*n* = 3), living in aged care (*n* = 1), having access to an emergency button (*n* = 1), a greater number of comorbidities (*n* = 1), higher education (*n* = 1), and using refreshments (*n* = 1). Though sex was explored in many studies, no clear patterns between risk of heatwave morbidity or mortality with sex were established in this review [33–35, 42, 45, 51, 59, 63–66, 68]. There were four identified risk factors relating to exposure, 10 risk factors relating to sensitivity, and seven risk factors relating to adaptive capacity. There was one identified protective factor relating to sensitivity, and eight relating to adaptive capacity. (Table 4).

**Table 4:** Significant risk factors and protective factors for heatwave morbidity and mortality for studies exploring all ages (*n* = 21)

Health Service	Risk Factors		Protective factors	
	Factor	References	Factor	References
Hospital Admissions	Low vegetation (S,AC)	[50, 52]	Living in aged care (AC)	[65]
	Low socioeconomic status (S, AC)	[65, 70]	Higher number of co-morbidities^a^ (S)	[65]
	Rural dwelling (E, S, AC)	[70]	Air conditioning in the bedroom (AC)	[66]
	Older adults (S)	[70]	Higher level of education (AC)	[66]
	Receiving community supports (AC)	[65]	Having an emergency button (AC)	[66]
	Living alone (AC)	[65]	Using refreshment (AC)	[66]
	No private insurance (AC)	[65]	Having more social activities (AC)	[66]
	Prior fall (S)	[65]	–	
	Children (S)	[68, 70, 77]	–	
ED Presentations	Children (S)	[68, 70, 77]	**–**	
	Older adults (S)	[36, 37, 40, 41, 51, 52, 68–70, 73, 74, 77]	**–**	
	Low socioeconomic status (S, AC)	[36, 70]	**–**	
	Rural dwelling (E, S, AC)	[70, 71]	**–**	
	Aboriginal and Torres Strait Islander status (S)	[68]	**–**	
	Pollutant exposure (S, AC)	[68]	**–**	
Ambulance Call- Outs	Older adults (S)	[40, 67, 74]	**–**	
	Low socioeconomic status (S, AC)	[67]	**–**	
	Pollutant exposure (S, AC)	[67]	**–**	
	Holiday period (E)	[67]	**–**	
	Coastal dwelling (E, S, AC)	[67]	**–**	
Mortality	Older adults (S)	[41, 51, 53, 74, 76, 78]	Air conditioning in bedroom (AC)	[64]
	Living alone (AC)	[64]	Social activity (AC)	[64]
	Urban dwelling (E, S, AC)	[71]	–	

Abbreviations: *E* Exposure, *S* Sensitivity, *AC* Adaptive capacity, *ED *Emergency Department

^a^The authors identified that a higher number of co-morbidities was a protective factor, and may be explained by increased health literacy, recurrent access to medical services, or being cared for by health practitioners or family members [73]

## Discussion

This systematic review demonstrates that heatwaves impact health, and subsequently health services, with increased demand seen ranging from ambulance services through to deaths Australia-wide. Climate change is increasing the frequency and intensity of heat events, and is worsening impacts on people, their communities, property, and the environment [6]. Disentangling the impact of heatwaves on health systems in Australia is complicated by the evolving definitions of heatwaves. The EHF, a formula first used by the Bureau of Meteorology, was cited frequently in Australian heatwave literature as a superior measure for determining the dose-response relationship between heatwaves and health service demand [31], as it allows for acclimitisation [33–36, 42, 43, 63, 71]. Notwithstanding, the results of this review highlight the complexities of heat-health relationships, along with differences in health outcomes for various medical conditions and vulnerable population sub-groups.

### Medical conditions

Individuals with chronic or mental health conditions are more susceptible to extreme heat. Increased risk of death and heatwave morbidity was found for those with cardiovascular, respiratory, renal, and neurological, mental, and behavioural disorders. Direct effects of heat, such as heat stroke, dehydration, organ failure, and cardiac arrest, can be exacerbated for medically dependent individuals [9]. However, Zhang et al. [73] found that a greater number of comorbidities had a protective effect for hospital admissions during heatwaves and posit that this may be due to greater interactions with the health system, increased health literacy, or being tended for by caretakers. This effect was not determined for other health services in the literature and should be investigated further.

### Vulnerable groups

In Australia, the major consequences of climate change will mainly impact susceptible population subgroups [6, 9]. The results indicate that heatwaves impact health service demand across all ages, however, we note that some ages and population groups are more impacted than others, such as children and the elderly. The social determinants of health contribute to heat susceptibility [79], and a person’s built environment [62, 63], socioeconomic status [35, 42, 51, 73], living arrangements [73], and welfare status [73] were identified as risk factors for health service presentations during heatwaves. It was not clear which risk factors were the most influential and this requires further investigation for prevention efforts. Managing the risks of heatwaves in Australia will require investment to reduce exposure and sensitivity and increase adaptive capacity, requiring multisectoral engagement including but not limited to the health sector, human services, infrastructure and transport.

There is considerable spatial variation is heatwave vulnerability across Australia [18]. A recent report by the Physical Environment Analysis Network (PEAN) highlighted significant variations in heatwave morbidity and mortality from neighbourhood to neighbourhood in Melbourne [18]. Similarly, a gradient of heatwave impacts have been identified across communities in Adelaide in terms of mortality and ambulance call-outs [24, 80]. Thus, community-level factors (e.g. housing quality, language and culture, socioeconomic status) must be considered for the development of effective heatwave preparedness plans [18, 81].

The urban heat island effect suggests that those who live in big cities with less greenspace are exposed to higher levels of heat [9]. Contrarily, metropolitan areas are known to have better heat adaptive capacity due to resources such as air-conditioning and having the ability to avoid outdoor activity [36, 61, 82, 83]. Rural residents may live with other disadvantages such as lower socioeconomic status and limited access to healthcare which impact their vulnerability to heatwaves [61, 82, 83]. A Queensland-based study determined similar impacts of heatwaves on emergency department visits for both rural and metropolitan dwellers [61]. In New South Wales, it was found that the impact of heatwaves on ED presentations was greater for inner and outer regional dwellers with little evidence of an effect in major cities [36]. However, intense heatwaves largely impact the risk of mortality in major cities, with a non-significant effect found in inner and outer regional and remote areas [36]. A similar effect has been found regarding the impact of heatwaves on general practice, where there was a disproportional impact on general practice demand depending on area-level factors [84]. Thus, the impacts of heatwaves on rural and metropolitan populations need to be explored separately so that local systems can plan accordingly and acknowledge the constraints and opportunities for operationalisation within the health service system.

### Impact on health services

Though this review explored the impact of heatwaves on health systems in terms of demand, health service resources are also impacted by extreme heat and may include staff, beds, power and water consumption and quality, and other infrastructure and mechanical failures [9, 85]. Emergency services need to be stocked in advance with appropriate cooling resources and equipment such as drinking water, ice, water spray bottles, fans, and cooling blankets [86]. Ambulance services may be challenged during heatwaves by pavement failure, buckling railway lines, and power losses [9]. As the frequency of heatwaves increase, local services must assess resourcing needs based on the demographic make-up of their population along with prevalence of medical conditions in the community.

### Heatwave preparedness in Australia

The recent Royal Commission into National Natural Disaster Arrangements highlighted that concurrent and consecutive hazard events are likely to be expected in Australia [87]. The implications of future heatwaves on health system capacity warrant evidence-based public health contingency planning and system integration. Creating health system resilience to cope with fluctuating demand is dependent on multiple systems and stakeholders [87]. The Royal Commission highlights the need for unity and unbroken linkage from federal, state and territory governments, down to individuals in communities [87]. Further, the evaluation of heatwave interventions is critical for the development of effective prevention strategies [70]. Nitschke et al. [70] found that heatwave morbidity was significantly decreased following the rollout of a heatwave warning system in Adelaide, South Australia. Future research should evaluate prevention strategies aimed at promoting disaster resilience Australia wide to identify opportunities for improvement [87].

### Air pollution and humidity

Pollutants commonly controlled for in Australian heatwave literature include ozone (O_3_), nitrogen dioxide (NO_2_), particulate matter with < 10 μm in aerodynamic diameter (PM_10_), and particulate matter with < 2.5 μm in aerodynamic diameter (PM_2.5_) [38, 39, 41, 52–54]. Disentangling how air pollution contributes to heatwaves and how heatwaves can further exacerbate the impacts of pollution is important but beyond the scope of this paper.

Relative humidity is an importance consideration as it is well established that the presence of humidity during heatwaves raises heat stress [20, 34, 88]. Though the EHF does not explicitly incorporate humidity, high humidity tends to result in high temperatures, which would be reflected in the calculation of this metric [20]. It is noted that heatwave temperatures may be more severe in tropical regions of Australia where higher humidity is present [88]. However, this review only identified one study which investigated heatwaves and health systems in tropical cities [61] (Townsville and Cairns, Queensland) and further explorations of heatwaves in these regions are required.

### Strengths and limitations

This is the first Australia-wide systematic review exploring the impacts of heatwaves on health system demand. The evidence collected in this review will be used for the development of future heatwave studies and preventive measures. This review has limitations. We explored morbidity and mortality for prolonged periods of heat of two or more consecutive days. Thus, studies exploring morbidity and mortality for hourly associations of heat and extremely hot days were not included. Heatwave data may not capture the whole picture of heat-health relationships [89]. Though exploring mortality was an outcome of this study, mortality was not included as a search term and some articles may not have been captured. Hospital admissions, emergency presentations, ambulance call outs, and mortality may report overlapping cases in studies reporting more than one of these outcomes. Additionally, this review did not include studies which analysed heatwave impacts on general practice or allied health, which are topics recommended for future studies. Another limitation was that a range of definitions were used for defining heatwaves, restricting the ability to conduct a meaningful synthesis of the results via meta-analysis.

The authors of this review recommend the use of the EHF for future heatwave research, as it has exhibited the most consistent dose-response relationship [31]. It is important to determine which methodology is most appropriate to detect the effects of heatwaves on the health of Australians. Inconsistencies and uncertainties between methodology across heatwave literature is noted in this review and elsewhere in the literature, impacting the ability to calculate a pooled estimate to determine the effect of heatwaves on morbidity and mortality [85]. Future studies should explore linked datasets to improve the understanding of how individuals move through the health system during heatwave periods. Further, many studies included in this review have established correlation between heatwaves and increases in health system demand but do not establish causal relationships. It is unknown if the conditions explored in this review occur due to or are exacerbated by heatwaves; hence the onset of occurrence is not always well understood using health system data. Well-designed empirical studies surrounding the impacts of heatwaves are needed.

## Conclusions

Heatwaves are predicted to increase in number, intensity, and duration. As such, we need to prepare a resilient health system which is able to meet the increased demand which occurs during heatwaves. This review shows that all health services see increased usage during heatwaves and notes specific conditions that are impacted by heatwaves. Vulnerable population subgroups are more likely to access health services during heatwaves. Evidence-based heatwave prevention programs should be developed and evaluated to address areas of opportunity related to sensitivity, exposure, and adaptive capacity. To help understand the benefits of any prevention programs, there needs to be a consistent definition of heatwaves used. The authors of this study recommend the use of the EHF for future studies in Australia.

## Supplementary Information

**Additional file 1.**

**Additional file 2.**

**Additional file 3.**

**Additional file 4.**

**Additional file 5.**

## Data Availability

All data is publicly available and is summarised in Supplementary File 2.
