# Supplementary material for: Systematic review of the impact of heatwaves on health service demand in Australia
Source: BMC Health Serv Res. 2022 Jul 28;22:960. doi: 10.1186/s12913-022-08341-3 (PMC9336006; doi:10.1186/s12913-022-08341-3)
Supplement: Supplementary file 1 — Additional file 1. [file 12913_2022_8341_MOESM1_ESM.docx]

Supplementary File 1. Search Strategy

| Database | # | Searches | Results |
| --- | --- | --- | --- |
| Medline | 1 | (heatwave* or "heat wave" or "heat-wave" or "heat waves" or "heat-waves").mp. [mp=title, abstract, original title, name of substance word, subject heading word, floating sub-heading word, keyword heading word, organism supplementary concept word, protocol supplementary concept word, rare disease supplementary concept word, unique identifier, synonyms] | 2,268 |
|  | 2 | ("Extreme Heat" or "Heat Stress" or "Heat Cramp" or "Heat Cramps").mp. [mp=title, abstract, original title, name of substance word, subject heading word, floating sub-heading word, keyword heading word, organism supplementary concept word, protocol supplementary concept word, rare disease supplementary concept word, unique identifier, synonyms] | 15,576 |
|  | 3 | extreme heat/ or temperature/ or hot temperature/ | 354,844 |
|  | 4 | 1 or 2 or 3 | 363,990 |
|  | 5 | Ambulances/ | 6,195 |
|  | 6 | ambulanc*.mp. [mp=title, abstract, original title, name of substance word, subject heading word, floating sub-heading word, keyword heading word, organism supplementary concept word, protocol supplementary concept word, rare disease supplementary concept word, unique identifier, synonyms] | 15,963 |
|  | 7 | 5 or 6 | 15,963 |
|  | 8 | 4 and 7 | 120 |
|  | 9 | Emergency Service, Hospital/ | 71,543 |
|  | 10 | emergency department.mp. [mp=title, abstract, original title, name of substance word, subject heading word, floating sub-heading word, keyword heading word, organism supplementary concept word, protocol supplementary concept word, rare disease supplementary concept word, unique identifier, synonyms] | 88,494 |
|  | 11 | 9 or 10 | 122,770 |
|  | 12 | 4 and 11 | 402 |
|  | 13 | Queensland.mp. [mp=title, abstract, original title, name of substance word, subject heading word, floating sub-heading word, keyword heading word, organism supplementary concept word, protocol supplementary concept word, rare disease supplementary concept word, unique identifier, synonyms] | 13,453 |
|  | 14 | 12 and 13 | 11 |
|  | 15 | 8 and 13 | 2 |
|  | 16 | Hospitals/ | 79,624 |
|  | 17 | hospital.mp. [mp=title, abstract, original title, name of substance word, subject heading word, floating sub-heading word, keyword heading word, organism supplementary concept word, protocol supplementary concept word, rare disease supplementary concept word, unique identifier, synonyms] | 1,284,877 |
|  | 18 | 16 and 17 | 46,258 |
|  | 19 | 16 or 17 | 1,318,243 |
|  | 20 | 4 and 19 | 2,545 |
|  | 21 | 13 and 20 | 13 |
|  | 22 | 14 or 15 or 21 | 17 |
|  | 23 | 8 or 12 or 20 | 2,700 |
|  | 24 | limit 23 to (english language and yr="2000 -Current") | 1,749 |
| Web of Science | 1 | TS=("extreme heat" OR "heat effects" OR  "temperature effects" OR "high temperature" OR "extreme temperature")  *Indexes=SCI-EXPANDED, SSCI, A&HCI, CPCI-S, CPCI-SSH, ESCI, CCR-EXPANDED, IC Timespan=2000-2021* | 282,851 |
|  | 2 | TOPIC: ("heat stress" or "heat stresses" or "heat cramp" or "heat cramps")  *Indexes=SCI-EXPANDED, SSCI, A&HCI, CPCI-S, CPCI-SSH, ESCI, CCR-EXPANDED, IC Timespan=All years* | 23,199 |
|  | 3 | #2 OR #1  *Indexes=SCI-EXPANDED, SSCI, A&HCI, CPCI-S, CPCI-SSH, ESCI, CCR-EXPANDED, IC Timespan=All years* | 302,336 |
|  | 4 | TOPIC: (ambulanc* or "ambulance services" OR "ambulance service" OR "ambulances")  *Indexes=SCI-EXPANDED, SSCI, A&HCI, CPCI-S, CPCI-SSH, ESCI, CCR-EXPANDED, IC Timespan=All years* | 9,049 |
|  | 5 | #4 AND #3  *Indexes=SCI-EXPANDED, SSCI, A&HCI, CPCI-S, CPCI-SSH, ESCI, CCR-EXPANDED, IC Timespan=All years* | 52 |
|  | 6 | TOPIC: ("emergency service" or "emergency services" or "emergency department" or "emergency departments" or "hospital" or "hospitals")  *Indexes=SCI-EXPANDED, SSCI, A&HCI, CPCI-S, CPCI-SSH, ESCI, CCR-EXPANDED, IC Timespan=All years* | 899,843 |
|  | 7 | #6 AND #3  *Indexes=SCI-EXPANDED, SSCI, A&HCI, CPCI-S, CPCI-SSH, ESCI, CCR-EXPANDED, IC Timespan=All years* | 609 |
|  | 8 | #7 OR #5  Refined by:  PUBLICATION YEARS: ( 2021 OR 2009 OR 2020 OR 2008 OR 2019 OR 2007 OR 2018 OR 2006 OR 2017 OR 2005 OR 2016 OR 2004 OR 2015 OR 2003 OR 2014 OR 2002 OR 2013 OR 2001 OR 2012 OR 2000 OR 2011 OR 2010 ) AND  LANGUAGES: ( ENGLISH )  *Indexes=SCI-EXPANDED, SSCI, A&HCI, CPCI-S, CPCI-SSH, ESCI, CCR-EXPANDED, IC Timespan=All years* | 601 |
|  | 9 | TOPIC: (Queensland)  *Indexes=SCI-EXPANDED, SSCI, A&HCI, CPCI-S, CPCI-SSH, ESCI, CCR-EXPANDED, IC Timespan=All years* | 20,998 |
|  | 10 | #8 AND #9  *Indexes=SCI-EXPANDED, SSCI, A&HCI, CPCI-S, CPCI-SSH, ESCI, CCR-EXPANDED, IC Timespan=All years* | 1 |
| Science Direct | 1 | (heatwave OR "heat wave") AND ("emergency department" OR hospital OR ambulance) limit to 2000-2021 | 1820 |
| PsychInfo | 1 | ("extreme heat" or "heat stress" or "heat stresses" or "heat cramp" or "heat cramps") OR (heatwave* or "heat wave" or "heat-wave" or "heat waves" or "heat-waves") OR mainsubject.Exact("extreme heat" OR "heat effects" OR "temperature perception" OR "temperature effects" OR "temperature") | 5,936 |
|  | 2 | ambulanc* OR mainsubject.Exact("ambulances") | 1,067 |
|  | 3 | [(("extreme heat" OR "heat stress" OR "heat stresses" OR "heat cramp" OR "heat cramps") OR mainsubject.Exact("extreme heat" OR "heat effects" OR "temperature perception" OR "temperature effects" OR "temperature")) AND (ambulanc* OR mainsubject.Exact("ambulances"))](https://search-proquest-com.elibrary.jcu.edu.au/recentsearches.recentsearchtabview.recentsearchesgridview.scrolledrecentsearchlist.checkdbssearchlink:rerunsearch/5C3CF150F0B844B4PQ/None?site=psycinfo&t:ac=RecentSearches) | 5 |
|  | 4 | ("emergency service" or "emergency services" or "emergency department" or "emergency departments" or "hospital" or "hospitals") OR mainsubject.Exact("emergency medical services" OR "hospital admission" OR "hospitals" OR "emergency services" OR "emergency service, hospital") | 432,648 |
|  | 5 | (("extreme heat" OR "heat stress" OR "heat stresses" OR "heat cramp" OR "heat cramps") OR mainsubject.Exact("extreme heat" OR "heat effects" OR "temperature perception" OR "temperature effects" OR "temperature")) AND (("emergency service" OR "emergency services" OR "emergency department" OR "emergency departments" OR "hospital" OR "hospitals") OR mainsubject.Exact("emergency medical services" OR "hospital admission" OR "hospitals" OR "emergency services" OR "emergency service, hospital")) | 405 |
|  | 6 | ((("extreme heat" OR "heat stress" OR "heat stresses" OR "heat cramp" OR "heat cramps") OR mainsubject.Exact("extreme heat" OR "heat effects" OR "temperature perception" OR "temperature effects" OR "temperature")) AND (ambulanc* OR mainsubject.Exact("ambulances"))) OR ((("extreme heat" OR "heat stress" OR "heat stresses" OR "heat cramp" OR "heat cramps") OR mainsubject.Exact("extreme heat" OR "heat effects" OR "temperature perception" OR "temperature effects" OR "temperature")) AND (("emergency service" OR "emergency services" OR "emergency department" OR "emergency departments" OR "hospital" OR "hospitals") OR mainsubject.Exact("emergency medical services" OR "hospital admission" OR "hospitals" OR "emergency services" OR "emergency service, hospital"))) | 408 |
|  | 7 | [((("extreme heat" OR "heat stress" OR "heat stresses" OR "heat cramp" OR "heat cramps") OR mainsubject.Exact("extreme heat" OR "heat effects" OR "temperature perception" OR "temperature effects" OR "temperature")) AND (ambulanc* OR mainsubject.Exact("ambulances"))) OR ((("extreme heat" OR "heat stress" OR "heat stresses" OR "heat cramp" OR "heat cramps") OR mainsubject.Exact("extreme heat" OR "heat effects" OR "temperature perception" OR "temperature effects" OR "temperature")) AND (("emergency service" OR "emergency services" OR "emergency department" OR "emergency departments" OR "hospital" OR "hospitals") OR mainsubject.Exact("emergency medical services" OR "hospital admission" OR "hospitals" OR "emergency services" OR "emergency service, hospital")))](https://search-proquest-com.elibrary.jcu.edu.au/recentsearches.recentsearchtabview.recentsearchesgridview.scrolledrecentsearchlist.checkdbssearchlink:rerunsearch/A64A91EC50D64460PQ/None?site=psycinfo&t:ac=RecentSearches)  *Limits= English, Year=2000-Current, Peer Review* | 344 |
|  | 8 | QUEENSLAND | 26,603 |
|  | 9 | ((((("extreme heat" OR "heat stress" OR "heat stresses" OR "heat cramp" OR "heat cramps") OR mainsubject.Exact("extreme heat" OR "heat effects" OR "temperature perception" OR "temperature effects" OR "temperature")) AND (ambulanc* OR mainsubject.Exact("ambulances"))) OR ((("extreme heat" OR "heat stress" OR "heat stresses" OR "heat cramp" OR "heat cramps") OR mainsubject.Exact("extreme heat" OR "heat effects" OR "temperature perception" OR "temperature effects" OR "temperature")) AND (("emergency service" OR "emergency services" OR "emergency department" OR "emergency departments" OR "hospital" OR "hospitals") OR mainsubject.Exact("emergency medical services" OR "hospital admission" OR "hospitals" OR "emergency services" OR "emergency service, hospital")))) AND (la.exact("ENG") AND pd(20000101-20210108) AND PEER(yes))) AND QUEENSLAND | 3 |
| Scopus | 1 | TITLE-ABS-KEY ( "extreme heat"  OR  "heat effects"  OR  "temperature effects"  OR  "temperature" ) | 5,108,375 |
|  | 2 | TITLE-ABS-KEY ( "heat stress"  OR  "heat stresses"  OR  "heat cramp"  OR  "heat cramps" ) | 29,203 |
|  | 3 | TITLE-ABS-KEY ( "extreme heat"  OR  "heat effects"  OR  "temperature effects"  OR  "temperature" ) )  OR  ( TITLE-ABS-KEY ( "heat stress"  OR  "heat stresses"  OR  "heat cramp"  OR  "heat cramps" ) ) | 5,118,916 |
|  | 4 | TITLE-ABS-KEY ( ambulanc*  OR  "ambulance services"  OR  "ambulance service"  OR  "ambulances" ) | 23,158 |
|  | 5 | ( ( TITLE-ABS-KEY ( "extreme heat"  OR  "heat effects"  OR  "temperature effects"  OR  "temperature" ) )  OR  ( TITLE-ABS-KEY ( "heat stress"  OR  "heat stresses"  OR  "heat cramp"  OR  "heat cramps" ) ) )  AND  ( TITLE-ABS-KEY ( ambulanc*  OR  "ambulance services"  OR  "ambulance service"  OR  "ambulances" ) ) | 571 |
|  | 6 | TITLE-ABS-KEY ( "emergency service"  OR  "emergency services"  OR  "emergency department"  OR  "emergency departments"  OR  "hospital"  OR  "hospitals" ) | 2,119,685 |
|  | 7 | ( ( TITLE-ABS-KEY ( "extreme heat"  OR  "heat effects"  OR  "temperature effects"  OR  "temperature" ) )  OR  ( TITLE-ABS-KEY ( "heat stress"  OR  "heat stresses"  OR  "heat cramp"  OR  "heat cramps" ) ) )  AND  ( TITLE-ABS-KEY ( "emergency service"  OR  "emergency services"  OR  "emergency department"  OR  "emergency departments"  OR  "hospital"  OR  "hospitals" ) ) | 24,707 |
|  | 8 | ( ( ( TITLE-ABS-KEY ( "extreme heat"  OR  "heat effects"  OR  "temperature effects"  OR  "temperature" ) )  OR  ( TITLE-ABS-KEY ( "heat stress"  OR  "heat stresses"  OR  "heat cramp"  OR  "heat cramps" ) ) )  AND  ( TITLE-ABS-KEY ( ambulanc*  OR  "ambulance services"  OR  "ambulance service"  OR  "ambulances" ) ) )  OR  ( ( ( TITLE-ABS-KEY ( "extreme heat"  OR  "heat effects"  OR  "temperature effects"  OR  "temperature" ) )  OR  ( TITLE-ABS-KEY ( "heat stress"  OR  "heat stresses"  OR  "heat cramp"  OR  "heat cramps" ) ) )  AND  ( TITLE-ABS-KEY ( "emergency service"  OR  "emergency services"  OR  "emergency department"  OR  "emergency departments"  OR  "hospital"  OR  "hospitals" ) ) ) | 24,984 |
|  | 9 | TITLE-ABS-KEY ( queensland ) | 42,741 |
|  | 10 | ( ( ( ( TITLE-ABS-KEY ( "extreme heat"  OR  "heat effects"  OR  "temperature effects"  OR  "temperature" ) )  OR  ( TITLE-ABS-KEY ( "heat stress"  OR  "heat stresses"  OR  "heat cramp"  OR  "heat cramps" ) ) )  AND  ( TITLE-ABS-KEY ( ambulanc*  OR  "ambulance services"  OR  "ambulance service"  OR  "ambulances" ) ) )  OR  ( ( ( TITLE-ABS-KEY ( "extreme heat"  OR  "heat effects"  OR  "temperature effects"  OR  "temperature" ) )  OR  ( TITLE-ABS-KEY ( "heat stress"  OR  "heat stresses"  OR  "heat cramp"  OR  "heat cramps" ) ) )  AND  ( TITLE-ABS-KEY ( "emergency service"  OR  "emergency services"  OR  "emergency department"  OR  "emergency departments"  OR  "hospital"  OR  "hospitals" ) ) ) )  AND  ( TITLE-ABS-KEY ( queensland ) ) | 52 |
|  | 11 | ( ( ( TITLE-ABS-KEY ( "extreme heat" OR "extreme temperature" OR "heat effects" OR "temperature effects" OR "high temperature" ) ) OR ( TITLE-ABS-KEY ( "heat stress" OR "heat stresses" OR "heat cramp" OR "heat cramps" ) ) ) AND ( TITLE-ABS-KEY ( ambulanc* OR "ambulance services" OR "ambulance service" OR "ambulances" ) ) ) OR ( ( ( TITLE-ABS-KEY ( "extreme heat" OR "heat effects" OR "temperature effects" OR "high temperature" OR "extreme temperature" ) ) OR ( TITLE-ABS-KEY ( "heat stress" OR "heat stresses" OR "heat cramp" OR "heat cramps" ) ) ) AND ( TITLE-ABS-KEY ( "emergency service" OR "emergency services" OR "emergency department" OR "emergency departments" OR "hospital" OR "hospitals" ) ) ) AND ( LIMIT-TO ( PUBYEAR , 2021 ) OR LIMIT-TO ( PUBYEAR , 2020 ) OR LIMIT-TO ( PUBYEAR , 2019 ) OR LIMIT-TO ( PUBYEAR , 2018 ) OR LIMIT-TO ( PUBYEAR , 2017 ) OR LIMIT-TO ( PUBYEAR , 2016 ) OR LIMIT-TO ( PUBYEAR , 2015 ) OR LIMIT-TO ( PUBYEAR , 2014 ) OR LIMIT-TO ( PUBYEAR , 2013 ) OR LIMIT-TO ( PUBYEAR , 2012 ) OR LIMIT-TO ( PUBYEAR , 2011 ) OR LIMIT-TO ( PUBYEAR , 2010 ) OR LIMIT-TO ( PUBYEAR , 2009 ) OR LIMIT-TO ( PUBYEAR , 2008 ) OR LIMIT-TO ( PUBYEAR , 2007 ) OR LIMIT-TO ( PUBYEAR , 2006 ) OR LIMIT-TO ( PUBYEAR , 2005 ) OR LIMIT-TO ( PUBYEAR , 2004 ) OR LIMIT-TO ( PUBYEAR , 2003 ) OR LIMIT-TO ( PUBYEAR , 2002 ) OR LIMIT-TO ( PUBYEAR , 2001 ) OR LIMIT-TO ( PUBYEAR , 2000 ) ) AND ( LIMIT-TO ( LANGUAGE , "English" ) ) | 1,399 |
| Proquest | 1 | (((("extreme heat") OR mainsubject.Exact("extreme heat" OR "heat effects" OR "high temperature" OR "extreme temperature")) AND (ambulanc* OR mainsubject.Exact("ambulances"))) OR ((("extreme heat") OR mainsubject.Exact("extreme heat" OR "heat effects" OR "high temperature" OR "extreme temperature")) AND (("emergency service" OR "emergency services" OR "emergency department" OR "emergency departments" OR "hospital" OR "hospitals") OR mainsubject.Exact("emergency medical services" OR "hospital admission" OR "hospitals" OR "emergency services" OR "emergency service, hospital")))) AND (la.exact("ENG") AND pd(20000101-20210112) AND PEER(yes)) | 2,258 |
|  |  |  |  |
