# Supplementary material for: Systematic review of the impact of heatwaves on health service demand in Australia
Source: BMC Health Serv Res. 2022 Jul 28;22:960. doi: 10.1186/s12913-022-08341-3 (PMC9336006; doi:10.1186/s12913-022-08341-3)
Supplement: Supplementary file 4 — Additional file 4. [file 12913_2022_8341_MOESM4_ESM.docx]

Supplementary File 4. Summary of studies where data could not be extracted

| Hatvani-Kovacs et al (2016) Can the excess heat factor indicate heatwave-related morbidity?  This case study explores three different ways of calculating the Excess Heat Factor (EHF) and the relationship between EHF and heatwave-related excess morbidity between 2008 and 2014 using ambulance callouts and hospital admission data. Excess morbidity were termed morbidity anomalies using ambulance call-out data This case study was driven by the recognition that there is no universally adopted definition globally of heatwaves nor the metrics for determining the relative intensities of differences between locations. Excess Heat Factor is a metric that was proposed to remedy this inconsistency, taking into account some of the otherwise unaccounted for factors that influence human health impacts of heatwaves such as acclimatisation and length of the heatwave. Adelaide is a city which experiences some of the highest strength heatwaves in Australia and, relatedly, is more resilient to the impact of heatwaves than other locations suggesting some level of acclimatization. The three approaches for calculating EHF were due to using different approaches to calculating daily mean temperature (DMT) which is a substantial element in the EHF calculation. Various data smoothing, adjustments and removals such as public holidays occurred.  Definition used for heatwaves – three consecutive days with maximum temperature of 35 degrees Celsius or above.  Key Findings: Regression analyses looking at different heatwave definitions were calculated with the use of EHF at greater than 0°C^2^ for the extreme heatwave that occurred in January 2009 and the EHF at greater than 40°C^2^ both produced the most significant *R*^2^ results. Emergency hospital admissions less influenced by heatwaves in this case study but also noted in the article to be supported by other literature. Higher priority categories ascribed to ambulance callouts also indicated a strong relationship with Bureau of Meteorology (BOM) approach to calculating DMT in the EHF calculation approach. The use of the BOM DMT was the strongest connection in determining daily anomalies in ambulance call outs. Therefore, use of ambulance call-outs data and EHF using BOM DMT were found in this study to offer the highest predicative advantage for heatwaves and heat alerts. |
| --- |
| Hatvani-Kovacs et al (2016) Assessment of Heatwave Impacts  Urban heatwaves are a function of climate change and urban heat islands have implications for health, energy use and other things. In recognition of this, the study explored excess morbidity using ambulance callouts, electricity demand and consumption and water supply during heatwaves in Adelaide for the period 2008 to 2014. Various data smoothing, adjustments and exclusions (i.e. public holidays) were undertaken to clean the data. Linear regression analyses used.  Definition used for heatwaves - Three heatwave definitions used to explore which was the better predictor of heatwave days with excess impacts (Excess heat factor (EHF), Daily mean temperature (DMaxT) >35°C and three days in a row with DMaxT > 35°C).  Key Findings exploring longitudinal and seasonal changes: Associations between electricity consumption and weather were not found, water supply was strongly related to DMaxT and an association between annual average of daily morbidity and weather not found. Daily electricity power and water supply strong relationship with DMaxT and very weak association with daily morbidities. DMT was best measure for daily electricity consumption.  Key Findings after cleaning and smoothing dataset: Peak demand for electricity and water during heatwaves accounted for approximately one-fifth of yearly amounts on these two parameters. EHF was the best predictor of excess morbidity. Anomalies in electricity demand and water supply were strongly associated with DMaxT on days with positive EHF. Temperature thresholds of heatwave impacts using DMaxT demonstrated shifts from negative to positive daily anomalies at these temperature points– excess morbidity at 28°C, electricity demand 30°C, electricity consumption at 32°C and water supply above 30°C. It was noted energy demand and water supply were more dependent on weather than morbidity but there are issues such as water restrictions and power outages which might have influenced energy and water use. |
| Langlois et al (2013) Using the Excess Heat Factor (EHF) to predict the risk of heat related deaths  Study reviewed deaths during 23/1/2009 t0 10/2/2009 including 13 days (26/1 to 7/2) where temperature exceeded 35°C and eight days were exceeded 40°C. Comparison period 27/2/2008-23/3/2008 which included 12 days (3/3 to 17/3) where maximum air temperature above 35°C and three days were exceeded 40°C. Measurement outcome: heat related or natural cause of death as determined by pathologist and/or coroner with date of death determined by coroner  Definition used for heatwaves: Excess heat factor (EHF) provides numerical value for the environmental temperature load – result above zero indicates heatwave conditions and higher values indicate more extreme conditions. Severe heatwave is when EHF excess threshold of severity for a specific geographic location.  Key findings: 2009 event is top EHF recorded for Adelaide. Total deaths = 58 with first occurring one day into the heatwave. Peak of heat-related deaths occurred immediately after peak of EHF (of 44°C^2^ for three days). 2008 comparison period peak EHF was 36°C^2^ ten days into heatwave. There was heat related death which occurred after peak EHF. 2/15 days from 2008 event had EHF severity threshold versus 6/13 days for 2009 event. EHF can be conceptualized as temperature which deviates from normal for a location given it takes into account sustained maximums across both day and night temperatures (unrelenting) and heat stress which is heat in excess of recent averages. Important finding: Elevated EHF in Adelaide preceded fatalities by 2-3 days. |
| Xie et al (2014) Calculate excess mortality during heatwaves using Hilbert-Huang transform algorithm  Explored use of Hilbert-Huang Transform (HHT) algorithm typically used for analyzing non-linear and non-stationary time series data. This was deemed appropriate given the characteristics of excess mortality during heatwaves meets these criteria and removing choice of smoothing parameter that is typically applied to other approaches. Two mortality datasets used for Brisbane and Chicago to determine excess mortality due to heatwaves. Brisbane dataset period: 1/7/1996 – 30/6/2004 (heatwave events summer 2000 and 2004). Chicago dataset period (comparison dataset): 1/1/1995 – 30/6/2020 (heatwave event – 1995 with 465 deaths). Primary risk factor for regression analysis – daily maximum temperature. Basically, HHT decomposes the data and sifts it to create a meaningful time-frequency-energy description.  Key findings: Estimated excess mortality in Brisbane was 62 for the 20 day heatwave period (7/2-26/2/2004). Six of those days accounted for 52 excess mortality deaths. Chicago estimated excess mortality (11-18/7/1995) as 510. It was determined from the Mortality and Morbidity Weekly Report (MMWR) there were 465 recorded heat-related deaths in Chicago (period 11-27/7/1995). Regression analysis founds significant results across four models (20 day heatwave period maximum temperature (absolute temperature), 20 day heatwave period with non-trend (daily maximum temperature anomalies), summer season with 91 days (1/12/20003-29/2/2004; absolute temperature) and summer season with 91 days (same period) with non-trend (daily maximum temperature anomalies). Heatwaves period using excess maximum temperature had the strongest positive correlation of all. Article is primarily concerned with the demonstration of applying this algorithm and validity of its use for morbidity and mortality calculations. |
| Longden (2019) The impact of temperature on mortality across different climate zones  Explores temperature-mortality using meteorological climate zones for Australia. Concerned with the question of whether an increase in heat-related mortality is balanced by a reduction in cold-related mortality and the implications of this for climate change (direction and magnitude of climate related mortality). National data set used to explore temperature-related mortality and regional variations on the basis of different climate zones and influence of different reference temperatures (heat and cold – subsequent points will focus on results for heat). Time series data which uses ambient temperatures and mortality data matched using local government area (LGA). Data sources: Registries of Births, Deaths and Marriages and National Coronial Information System for mortality data with usual residential location postcode data used to determine LGA of residence (note – doesn’t imply died at this residence!). Mortality data from all causes excluding external causes– 1/1/2006 to 31/10/2017.Ambient temperature using data from 305 weather stations from the Bureau of Meteorology (BOM) using the address of the Local Government office for each LGA. Aggregated LGA-level daily average temperature data using mean for region. Thermal climate zones determined using BOM data with zones based on temperature and humidity. Comparison point for climatic zones is using Acclimitisation Excess Heat Index (EHI-A) which measures difference between three day temperature average compared to 30-day temperature average. EHI-A is one of the two components of excess heat factor (EHF) calculations. Other points of analysis: Geographical determination (capital city or rest/regional area) using Australian Bureau of Statistics (ABS) Statistical Area Level 4 (SA4) classifications. Also considered socio-economic index for areas (SEIFA) measures to determine socio-economic levels of advantage and disadvantage for locations from ABS. Multiple types of regression analyses undertaken to explore data by regions, sensitivity analysis and estimation of cumulative exposure-response associations. Regional, state/territory and national aggregations created.  Key findings: Use of minimum mortality temperature as the reference temperature shows more impact attributable to heat impacts than use of median in the cumulative exposure-response associations mapped by climate zones, geographical areas (although there are some differences in percentile fits between the two reference temperatures for this analysis) and SEIFA. The two highest attributable fractions to heat on mortality were for Brisbane and the Rest of Queensland accounting for 36.6% and 37.5%, respectively (using minimum mortality temperature as a reference of 11.8°C and 15.3°C, respectively). |
