# Supplementary material for: Systematic review of the impact of heatwaves on health service demand in Australia
Source: BMC Health Serv Res. 2022 Jul 28;22:960. doi: 10.1186/s12913-022-08341-3 (PMC9336006; doi:10.1186/s12913-022-08341-3)
Supplement: Supplementary file 5 — Additional file 5. [file 12913_2022_8341_MOESM5_ESM.docx]

Supplementary File 5. Significant effects of heatwaves on medical conditions and presentations in Australia

| Significant effects of heatwaves on medical conditions and presentations in Australia | | | | | |
| --- | --- | --- | --- | --- | --- |
| Health Service | Medical Condition | Age group | Heatwave Definition | Reference | Epidemiological measure (95% CI) |
| Hospital Admissions | Cardiovascular | All ages | >99^th^ percentile, 2+ days | Huang et al. (2012) | YLL= 85 (40, 129) |
|  |  | All ages | ≥40°C, 5 days | Zhang et al. (2016) | AOR=13.56 (1.27, 144.86) |
|  | Acute Myocardial Infarction | 35+ | 3-day avg > ≥27°C | Loughnan et al. (2010) | Increased by 37.7% |
|  | Ischaemic | 65-74 | ≥35°C, 3+ days | Nitschke et al. (2007) | IRR= 1.08 (1.01-1.15) |
|  | Alzheimer’s | 35+ | ≥95^th^ percentile, 2+ days | Xu et al. (2019) | Increased by 51% (2%. 126%) |
|  | Dementia | All ages | ≥35°C, 3+ days | Hansen et al. (2008) | IRR= 1.174 (1.017-1.355) |
|  |  | All ages | ≥40°C, 5 days | Zhang et al. (2016) | AOR= 26.43 (1.99, 350.73) |
|  | Mental and Behavioral Disorders | All ages | ≥35°C, 3+ days | Hansen et al. (2008) | IRR= 1.073 (1.0175, 1.132) |
|  |  | All ages | ≥35°C, 3+ days | Nitschke et al. (2007) | IRR= 1.07 (1.01, 1.13) |
|  |  | All ages | ≥35°C, 3+ days | Nitschke et al. (2011) | IRR= 1.05 (1.00, 1.10) |
|  |  | 65-74 | ≥35°C, 3+ days | Nitschke et al. (2011) | IRR= 1.12 (1.01, 1.24) |
|  |  | ≥75 | ≥35°C, 3+ days | Nitschke et al. (2011) | IRR= 1.10 (1.01, 1.19) |
|  |  | ≥75 | ≥35°C, 3+ days | Nitschke et al. (2007) | IRR= 1.17 (1.07, 1.28) |
|  | Organic, including symptomatic, mental disorders | All ages | ≥35°C, 3+ days | Hansen et al. (2008) | IRR= 1.213 (1.091, 1.349) |
|  | Mood (affective) disorders | All ages | ≥35°C, 3+ days | Hansen et al. (2008) | IRR= 1.091 (1.004, 1.185) |
|  | Neurotic, stress-related, and somatoform disorders | All ages | ≥35°C, 3+ days | Hansen et al. (2008) | IRR= 1.097 (1.018, 1.181) |
|  | Disorders of psychological development | All ages | ≥35°C, 3+ days | Hansen et al. (2008) | IRR= 1.641 (1.086, 2.480) |
|  | Senility | All ages | ≥35°C, 3+ days | Hansen et al. (2008) | IRR= 2.366 (1.200, 4.667) |
|  | Renal | All ages | ≥95^th^ percentile, 2+ days | Hansen et al. (2008) | IRR= 1.100 (1.003, 1.206) |
|  |  | All ages | ≥35°C, 3+ days | Nitschke et al. (2007) | IRR= 1.13 (1.03, 1.25) |
|  |  | All ages | ≥35°C, 3+ days | Nitschke et al. (2011) | IRR= 1.10 (1.01, 1.20) |
|  |  | All ages | ≥35°C, 3+ days | Nitschke et al. (2007) | IRR= 1.16 (1.04, 1.30) |
|  |  | All ages | ≥40°C, 5 days | Zhang et al. (2013) | OR= 1.72 (1.07, 2.94) |
|  |  | 5-14 | ≥35°C, 3+ days | Nitschke et al. (2011) | IRR= 2.64 (1.47, 4.73) |
|  |  | 15-64 | ≥95^th^ percentile, 2+ days | Hansen et al. (2008) | IRR= 1.130 (1.025, 1.247) |
|  |  | 15-64 | ≥35°C, 3+ days | Nitschke et al. (2011) | IRR= 1.13 (1.03, 1.25) |
|  |  | ≥75 | ≥35°C, 3+ days | Nitschke et al. (2011) | IRR= 1.23 (1.03, 1.47) |
|  |  | ≥75 | ≥35°C, 3+ days | Nitschke et al. (2011) | IRR= 1.48 (1.15, 1.88) |
|  | Acute renal failure | All ages | ≥95^th^ percentile, 2+ days | Hansen et al. (2008) | IRR= 1.255 (1.037, 1.519) |
|  | Urinary disease | All ages | EHF | Borg et al. (2019) | IRR= 1.090 (1.048, 1.133) |
|  | Kidney disease | All ages | EHF_Severe_ | Xiao et al. (2017) | RR= 1.157 (1.047, 1.279) |
|  | Acute kidney injury | All ages | EHF | Borg et al. (2019) | IRR= 1.335 (1.204, 1.480) |
|  | Direct heat | All ages | ≥35°C, 3+ days | Nitschke et al. (2011) | IRR= 3.12 (2.51, 3.87) |
|  |  | All ages | ≥35°C, 3+ days | Nitschke et al. (2011) | IRR= 2.62 (1.32, 5.20) |
|  |  | All ages | ≥35°C, 3+ days | Nitschke et al. (2011) | IRR= 13.66 (8.89, 20.98) |
|  |  | 0-4 | ≥35°C, 3+ days | Nitschke et al. (2011) | IRR= 2.13 (1.27, 3.57) |
|  |  | 15-64 | ≥35°C, 3+ days | Nitschke et al. (2011) | IRR= 2.59 (2.01, 3.33) |
|  |  | 15-64 | ≥35°C, 3+ days | Nitschke et al. (2011) | IRR= 2.53 (1.22, 5.25) |
|  |  | 15-64 | ≥35°C, 3+ days | Nitschke et al. (2011) | IRR= 11.53 (7.18, 18.53) |
|  |  | 65-74 | ≥35°C, 3+ days | Nitschke et al. (2011) | IRR= 3.05 (2.06, 4.54) |
|  |  | 65-74 | ≥35°C, 3+ days | Nitschke et al. (2011) | IRR= 7.06 (3.05, 16.30) |
|  |  | ≥75 | ≥35°C, 3+ days | Nitschke et al. (2011) | IRR= 3.65 (2.92, 4.57) |
|  |  | ≥75 | ≥35°C, 3+ days | Nitschke et al. (2011) | IRR= 3.05 (1.54, 6.06) |
|  |  | ≥75 | ≥35°C, 3+ days | Nitschke et al. (2011) | IRR=19.23 (12.44, 29.70) |
| Emergency Department | Cardiovascular | All ages | >95^th^ percentile, 2+ days | Toloo et al. (2014) | RR= 1.01 |
|  |  | All ages | 3-day max > 99^th^ percentile | Khalaj et al. (2010) | RO = 1.12 (1.08, 1.16) |
|  | Respiratory | All ages | >95^th^ percentile, 2+ days | Toloo et al. (2014) | RR= 1.02 |
|  |  | All ages | 3-day max > 99^th^ percentile | Khalaj et al. (2010) | RO = 1.14 (1.08, 1.21) |
|  | Asthma | All ages | 3-day max > 99^th^ percentile | Khalaj et al. (2010) | RO = 1.57 (1.40, 1.76) |
|  | COPD | All ages | 3-day max > 99^th^ percentile | Khalaj et al. (2010) | RO = 1.12 (1.01, 1.24) |
|  |  |  |  |  |  |
|  | Renal | All ages | ≥35°C, 2+ days | Wang et al. (2012) | OR=1.41 (1.09, 1.83) |
|  |  | All ages | 3-day max > 99^th^ percentile | Khalaj et al. (2010) | RO = 1.11 (1.05, 1.18) |
|  |  | All ages | ≥35°C, 3+ days | Nitschke et al. (2011) | IRR= 1.39 (1.26,1.54) |
|  |  | All ages | ≥35°C, 3+ days | Nitschke et al. (2016) | IRR= 1.39 (1.23, 1.58) |
|  |  | 0-14 | ≥37°C, 2+ days | Wang et al. (2014) | OR= 2.08 (1.05, 4.09) |
|  |  | 15-64 | ≥35°C, 3+ days | Nitschke et al. (2016) | IRR= 1.32 (1.16, 1.50) |
|  |  | 65-74 | ≥35°C, 2+ days | Wang et al. (2012) | OR= 2.25 (1.05, 4.83) |
|  |  | ≥75 | ≥35°C, 3+ days | Nitschke et al. (2016) | IRR= 1.67 (1.32, 2.13) |
|  | Urinary disease | All ages | EHF | Borg et al. (2019) | IRR= 1.046 (1.016, 1.076) |
|  | Urolithiasis | All ages | EHF | Borg et al. (2019) | IRR= 1.106 (1.046, 1.169) |
|  | Acute kidney injury | All ages | EHF | Borg et al. (2019) | IRR= 1.416 (1.258, 1.594) |
|  | Mental and behavioral disorders | All ages | 3-day max > 99^th^ percentile | Khalaj et al. (2010) | RO = 1.11 (1.06, 1.17) |
|  |  | All ages | ≥35°C, 3+ days | Nitschke et al. (2011) | IRR = 1.11 (1.04-1.18) |
|  | Endocrine, nutritional, and metabolic diseases | All ages | ≥ 95^th^ percentile, 3+ days | Xu et al. (2019) | RR= 1.18 (1.04, 1.34) |
|  | Diseases of the nervous system | All ages | ≥ 95^th^ percentile, 3+ days | Xu et al. (2019) | RR= 1.09 (1.02, 1.17) |
|  |  | All ages | 3-day max > 99^th^ percentile | Khalaj et al. (2010) | RO = 1.12 (1.04, 1.19) |
|  | Diseases of the genitourinary system | All ages | ≥ 95^th^ percentile, 3+ days | Xu et al. (2019) | RR= 1.05 (1.00, 1.09) |
|  | Neoplasm | All ages | 3-day max > 99^th^ percentile | Khalaj et al. (2010) | RO = 1.11 (1.02, 1.20) |
|  | Heat related | All ages | ≥35°C, 3+ days | Nitschke et al. (2011) | IRR= 2.68 (2.19, 3.28) |
|  |  | All ages | ≥35°C, 3+ days | Nitschke et al. (2011) | IRR= 12.01 (9.55, 15.12) |
|  |  | All ages | ≥35°C, 3+ days | Nitschke et al. (2016) | IRR= 12.03 (9.23, 15.68) |
|  |  | All ages | ≥35°C, 3+ days | Nitschke et al. (2016) | IRR= 5.27 (3.81, 7.30) |
|  |  | All ages | >95^th^ percentile, 2+ days | Toloo et al. (2014) | RR= 4.98 (3.88, 6.38) |
|  |  | 0-14 | >95^th^ percentile, 2+ days | Toloo et al. (2014) | RR= 3.00 (1.19, 7.56) |
|  |  | 15-64 | >95^th^ percentile, 2+ days | Toloo et al. (2014) | RR= 3.64 (2.56, 5.18) |
|  |  | 15-64 | ≥35°C, 3+ days | Nitschke et al. (2016) | IRR= 12.41 (8.69, 17.74) |
|  |  | 15-65 | ≥35°C, 3+ days | Nitschke et al. (2016) | IRR= 6.10 (4.02, 9.25) |
|  |  | 65-74 | >95^th^ percentile, 2+ days | Toloo et al. (2014) | RR= 7.29 (3.93, 13.53) |
|  |  | 65-74 | ≥35°C, 3+ days | Nitschke et al. (2016) | IRR= 9.48 (6.13, 14.65) |
|  |  | 65-74 | ≥35°C, 3+ days | Nitschke et al. (2016) | IRR= 4.41 (2.75, 7.06) |
|  |  | 75+ | >95^th^ percentile, 2+ days | Toloo et al. (2014 ) | RR= 9.17 (5.45, 15.44) |
|  |  | 75+ | ≥35°C, 3+ days | Nitschke et al. (2016) | IRR= 15.85 (12.49, 20.12) |
|  |  | 75+ | ≥35°C, 3+ days | Nitschke et al. (2016) | IRR= 6.12 (4.70, 7.97) |
| Ambulance | Cardiovascular | All ages | ≥35°C, 2+ days | Turner et al. (2013) | Added effect= 29.5% (0.4%, 67.0%) |
|  |  | All ages | ≥35°C, 3+ days | Nitschke et al. (2011) | IRR= 1.10 (1.01, 1.20) |
|  |  | All ages | ≥35°C, 3+ days | Nitschke et al. (2011) | IRR= 1.13 (1.03, 1.23) |
|  |  | 15-64 | ≥35°C, 3+ days | Nitschke et al. (2011) | IRR= 1.16 (1.01, 1.34) |
|  |  | 65-74 | ≥35°C, 2+ days | Turner et al. (2013) | Added effect= 163.7% (56.0%, 345.8%) |
|  | Respiratory | All ages | ≥35°C, 2+ days | Turner et al. (2013) | Added effect= 48.7% (6.4%, 107.7%) |
|  |  | 5-14 | ≥35°C, 3+ days | Nitschke et al. (2011) | IRR= 1.47 (1.13, 1.91) |
|  |  | 15-64 | ≥35°C, 3+ days | Nitschke et al. (2011) | IRR= 1.32 (1.08, 1.61) |
|  |  | 65-74 | ≥35°C, 2+ days | Turner et al. (2013) | Added effect= 127.3% (14.7%, 350.3%) |
|  | Neurological | 65-74 | ≥35°C, 3+ days | Nitschke et al. (2011) | IRR= 1.39 (1.01, 1.91) |
| Mortality | Cardiovascular | All ages | ≥35°C, 2+ days | Wang et al. (2012) | OR= 1.89 (1.44, 2.48) |
|  |  | All ages | >95^th^ percentile, 2+ days | Wang et al. (2015) (Brisbane) | RR= 1.09 (1.04, 1.15) |
|  |  | All ages | >95^th^ percentile, 2+ days | Wang et al. (2015) (Melbourne) | RR= 1.05 (1.02, 1.09) |
|  |  | All ages | >95^th^ percentile, 2+ days | Wang et al. (2015)  (Sydney) | RR= 1.06 (1.03, 1.08) |
|  |  | All ages | ≥40°C, 5 days | Zhang et al. (2017) | AOR= 22.4 (1.7, 303.0) |
|  |  | 65-74 | ≥35°C, 2+ days | Wang et al. (2012) | OR= 2.81 (1.21, 6.51) |
|  |  | 75+ | ≥35°C, 2+ days | Wang et al. (2012) | OR= 1.83 (1.35, 2.48) |
|  |  | 75+ | >95^th^ percentile, 2+ days | Wang et al. (2015) (Brisbane) | OR = 1.10 (1.05, 1.15) |
|  |  | 75+ | >95^th^ percentile, 2+ days | Wang et al. (2015) (Melbourne) | OR = 1.06 (1.03, 1.10) |
|  |  | 75+ | >95^th^ percentile, 2+ days | Wang et al. (2015) (Sydney) | OR = 1.08 (1.05, 1.11) |
|  | Diabetes | 75+ | ≥35°C, 2+ days | Wang et al. (2012) | OR= 9.96 (1.02, 96.85) |
|  | Mental and Behavioral Disorders | 65-74 | ≥35°C, 3+ days | Hansen et al. (2008) | IRR=2.395 (1.165, 4.922) |
|  | Alzheimer’s | 35+ | ≥95^th^ percentile, 2+ days | Xu et al. (2019) | Increased by 269% (76%. 665%) |
|  | Dementia | 15-64 | ≥35°C, 3+ days | Hansen et al. (2008) | IRR= 5.058 (1.205, 21.232) |
|  | Schizophrenia, schizotypal, and delusional disorders | All ages | ≥35°C, 3+ days | Hansen et al. (2008) | IRR = 2.079 (1.045, 4.138) |
